# Development of a Novel Vaccine Candidates against *Cardiobacterium valvarum* through Reverse Vaccinology and Computational Approaches

**DOI:** 10.1155/2023/6325568

**Published:** 2023-06-28

**Authors:** Faisal F. Albaqami, Ali Altharawi, Hassan N. Althurwi, Khalid M. Alharthy, Muhammad Tahir ul Qamar, Ziyad Tariq Muhseen, Madiha Iqbal

**Affiliations:** ^1^Department of Pharmacology, College of Pharmacy, Prince Sattam Bin Abdulaziz University, Al-Kharj 11942, Saudi Arabia; ^2^Department of Pharmaceutical Chemistry, College of Pharmacy, Prince Sattam Bin Abdulaziz University, Al-Kharj 11942, Saudi Arabia; ^3^Department of Bioinformatics and Biotechnology, Government College University Faisalabad (GCUF), Faisalabad 38000, Pakistan; ^4^Department of Pharmacy, Al-Mustaqbal University College, Hillah, Babylon 51001, Iraq; ^5^Department of Health and Biological Sciences, Abasyn University, Peshawar, Pakistan

## Abstract

Antibiotic resistance is a major public health concern that has resulted in high healthcare costs, increased mortality, and the emergence of novel bacterial diseases. *Cardiobacterium valvarum*, an antibiotic-resistant bacterium, is one of the leading causes of heart disease. Currently, there is no licensed vaccination against *C. valvarum*. In this research, an *in silico*-based vaccine was designed against *C. valvarum* using reverse vaccinology, bioinformatics, and immunoinformatics techniques. 4206 core proteins, 2027 nonredundant proteins, and 2179 redundant proteins were predicted. Among nonredundant proteins, 23 proteins were predicted in an extracellular membrane, 30 in the outer membrane, and 62 in the periplasmic membrane region. After applying several subtractive proteomics filters, two proteins, TonB-dependent siderophore receptor and hypothetical protein, were chosen for epitope prediction. In the epitope selection phase, B and T-cellepitopes were analyzed and shortlisted for vaccine design. The vaccine model was designed by linking selected epitopes with GPGPG linkers to avoid flexibility. Furthermore, the vaccine model was linked to cholera toxin B adjuvant to induce a proper immune response. The docking approach was utilized to analyze binding affinity to immune cell receptors. Molecular docking results predicted 12.75 kcal/mol for a Vaccine with MHC-I, 6.89 for a vaccine with MHC-II, and 19.51 vaccine with TLR-4. The MMGBSA estimated -94, -78, and -76 kcal/mol for TLR-4 and vaccine, MHC-I and vaccine, and MHC-II and vaccine, while the MMPBSA analysis estimated -97, -61, and -72 kcal/mol for TLR-4 with the vaccine, MHC-I with vaccine, and MHC-II with a vaccine. Molecular dynamic simulation analysis revealed that the designed vaccine construct has proper stability with immune cell receptors as it is essential for inducing an immune response. In conclusion, we observed that the model vaccine candidate has the potency to induce an immune response in the host. However, the study is designed purely on a computational basis; hence, experimental validation is strongly recommended.

## 1. Introduction

Bacterial infections are usually treated with antibiotics/medicines. Long-term usage of these medicines leads to antibiotic resistance [1]. Resistant bacteria become difficult to treat when compared to nonresistant bacteria, both in the case of animal or human infections. The consequences of antibiotic resistance include prolonged hospitalization, an increase in medical cost, and an increased mortality rate [2]. The prescription of medicines and usage of antibiotics needs to carry on with great care. Antibiotic resistance is a major threat if these practices are not changed [3]. Behavior modifications will require a change in lifestyles by requiring vaccinations, hand washing, safe sexual activity, and a good self-hygiene regime; infection sickness that affects the heart or blood vessels is referred to as a cardiovascular disease [4].

The elevated risk of blood clots and fat deposits in the arteries (atherosclerosis) is usually associated with cardiovascular disease. It is linked with arterial damage in different organs as well, for example, kidneys, brain, eyes, and heart [5]. Cardiovascular disease is one of the primary causes of death and disability in the United Kingdom, yet it may often be avoided by following a healthy lifestyle. Endocarditis is caused by Cardiobacterium valvarum, a recently discovered “*Haemophilus* species, *Aggregatibacter actinomycetemcomitans*, *Cardiobacterium hominis, Eikenella corrodens*, *and Kingella kingae*” commonly known as (HACEK) pathogen. In terms of culture, gram stain, and growth properties, these two species of *Cardiobacterium* are morphologically indistinguishable [6]. Under standard CO2 incubation conditions, isolates of C.valvarum on 5 percent sheep blood show optimal growth by day 3, but with limited growth on chocolate agar and sheep blood agar, colonies are nonhemolytic to mildly a-hemolytic. Using 16S PCR, the *Cardiobacterium* species can be differentiated [7]. *Cardiobacterium* is a fastidious gram-negative bacillus that is an infrequent human pathogen in therapeutic circumstances. *C. valvarum* and *C. hominis* are the two species of the genus *Cardiobacterium*, with the latter having a higher rate of infection. Due to its phenotypic characteristics, the clinical features of *C. valvarum* infection have not yet been thoroughly researched [7]. Since it can be challenging to identify *C. valvarum,* referral labs that use molecular identification techniques may be needed. The clinical features of *C. valvarum* endocarditis were evaluated in one of the case studies, which involved a 36-year-old man who had suffered an abrupt myocardial infarction and was found to have bicuspid aortic valve subacute bacterial endocarditis [8]. Vaccine discovery is a significant breakthrough that represents a more rational way to dealing with biomedical sciences [9]. Vaccines developed using conventional vaccinology have several disadvantages as compared to epitope-based chimeric vaccines. For example, they can replace many wet lab studies and save time because they are less expensive to manufacture and do not require microbial cultivation [10]. In this work, reverse vaccinology, immunoinformatics, and different biophysical approaches were utilized for vaccine target identification, epitope prediction, and prioritization and designing of multiepitope vaccine against the target pathogen. They are a safer alternative since they are highly specific and stable and do not contain full viruses [11]. Therefore, the approach used in computer-aided vaccine designs is a better reciprocal choice that will expedite, shorten, and increase efficacy [12]. The study is mainly aimed at designing of multiepitope vaccine construct against *C. valvarum* by using several immunoinformatics and reverse vaccinology approaches.

## 2. Research Methodology

**Figure 1:**
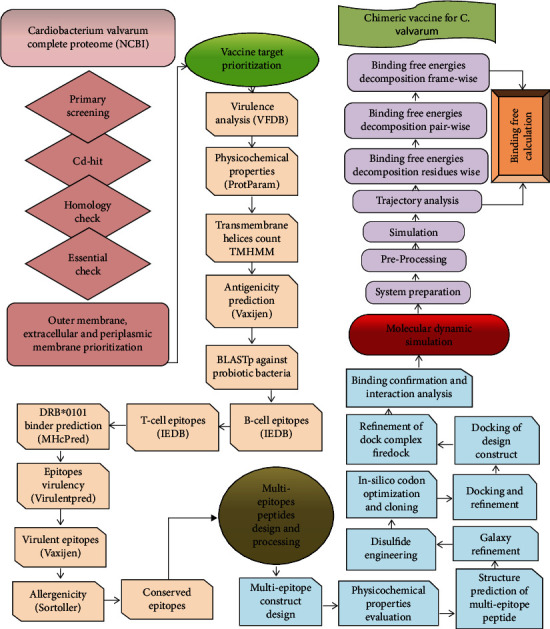
Schematic representation of research methodology.

The following are the main steps used in the methodology to design the multiepitope vaccine against *C. valvarum* as shown in the flow chart mentioned in Figure 1.

### 2.1. Proteome Retrieval of *C. valvarum* and Subtraction of Core Proteins

In this step, the whole complete proteome of *C. valvarum* was retrieved from the “National Center for Biotechnology Information” database (NCBI) (https://www.ncbi.nlm.nih.gov/) and converted into FASTA format [13]. Core sequences were predicted through Bacterial Pan-Genome Analysis (BPGA) (https://iicb.res.in/bpga/) [14]. Core protein was subjected to subtractive bioinformatics analysis to find a good vaccine candidate. These are computational-based approaches to select vaccine targets by excluding the nonessential proteins from the vaccine construct. In the first step, the paralogous proteins were removed (that were added in CD/hit analysis) (https://sites.google.com/view/cd-hit) [15]. The nonsimilar proteins were considered for surface localization which was checked by PSORTb (https://www.psort.org/psortb/server) [16], and virulent protein sequence was analyzed in Virulence Factor Database (VFDB) (https://www.psort.org/psortb/analysis) [17]. In addition, using TMHMM 2.0 tool (https://services.healthtech.dtu.dk/services/TMHMM-2.0/) predicts the transmembrane helices with greater than 1 cut-off value [18]. Vaxijen 2.0 (http://www.ddg-pharmfac.net/vaxijen/VaxiJen/VaxiJen.html) online web server was used for the analysis of antigenicity with a threshold value of 0.5 [19]. Furthermore, to check the allergenicity of the protein, Allertop 2.0 was used [20]. Next, physiochemical properties were analyzed by the protparam tool (https://web.expasy.org/cgi-bin/protparam/protparam) [21]. Next, to check the analysis against human (taxid: 9606) and 3 Lactobacillus species: “*L. rhamnosus* (taxid: 47715), *L. johnsonii* (taxid: 33959), and *L. casei* (taxid: 1582),” homology analysis was done through BLASTp (https://blast.ncbi.nlm.nih.gov/Blast.cgi) [22]. The shortlisted proteins were further subjected to epitope prediction.

### 2.2. Epitope Prediction Phase

In this phase, B-cell epitopes and T-cell epitopes were predicted from the target proteins using the immune epitope database and analysis resource IEDB tool (https://www.iedb.org/) [23]. This prediction is pivotal to obtain a humoral and cellular response against the antigen. BepiPred 2.0 [24] was utilized to predict the B- cell linear epitopes. Furthermore, the predicted B-cell epitopes were utilized to predict the T-cell epitope, and this procedure was completed successfully using the IEDB T-cell prediction tool, with epitopes prioritized based on low percentile score. In addition, the predicted epitopes having binding potency with the DRB × 0101 allele were analyzed using MHcPred tool [25]. Vaxijen 2.0, ToxinPred, InvivoGen, and Allertop 2.0 tools (https://www.ddg-pharmfac.net/AllerTOP/) were used to check their antigenicity, toxicity, allergenicity, and solubility [26], respectively. The shortlisted epitopes will then be used to design a vaccine.

### 2.3. Vaccine Construction Phase

To overcome the issue of a single peptide vaccine, the multiepitope vaccine was constructed, which consists not only of a single epitope but many different types of immune-dominant epitopes [27]. To evoke substantial immune responses, multipeptide vaccine construct was designed [28]. To create a multiepitope vaccine construct, GPGPG linkers were used to link the selected epitopes. Finally, the vaccine construct was linked to a good and safe adjuvant (Cholera Toxin-B Subunit) [29].

### 2.4. Physiochemical Properties of the Multiepitope Vaccine Construct

In this step, the multiepitope vaccine construct was checked for physiochemical properties through the online ProtParam tool (Expasy) web server [30]. The following properties of the designed vaccine were checked: molecular weight (MW); instability index (II); and aliphatic index (AI) [31].

### 2.5. Vaccine's Structure Modeling

The structure was predicted through the 3DPro tool using the sequence of vaccine construct [32]. For molecular recognition, the prediction of the stable vaccine structure is essential. An online Galaxy web server and Galaxy Refine 2 tool were used for loop modeling [33] and refinement, respectively, [34].

### 2.6. *In Silico* Cloning and Disulfide Engineering

Disulfide engineering was done by Design 2.0 webserver [35]. In this step, the vaccine candidate's structural stability was improved and the mutant structure was created by making disulfide bonds. In the *E. coli* system expression of the cloned vaccine sequence, codon optimization approach was used. In this approach, the multiepitope vaccine sequence is reversed to the DNA sequence using Java Codon Adaptation Tool (JCat) [36].

### 2.7. Molecular Docking and Refinement

The PATCHDOCK [37] and FIREDOCK [38] web servers were used to perform the molecular docking and refinement of docking results. Docking of the vaccine with “MHC-I, MHC-II, and TLR-4” receptors of the immune cells was performed [39]. The docked solutions of PATCHDOCK were submitted for refinement. Numerous steric clash errors of intermolecular conformational are removed using the FIREDOCK server from the solutions and reranked them [31].

### 2.8. Molecular Dynamic Simulation

The molecular dynamic simulation was applied for the assessment of the movement of docked molecules. In the molecular dynamic simulation, the complex was evaluated for 100 ns of time period using AMBER [40]. As a force field, FF14SB was used [41]. On the other hand, for the vaccine-receptor complex, the submersion “TIP3P3 water box (12 Angstrom)” was used [27]. Moreover, the SHAKE algorithm was used to constrain a hydrogen bond. Furthermore, these complexes were equilibrated and heated, and, afterwards, a production run was carried out for 100 ns [42].

### 2.9. Calculations for Binding-Free Energies

Binding-free energies of docked complexes were estimated through “MM/PBSA and MM/GBSA” available in AMBER20 [43]. With the help of the MMPBSA.py module of AMBER, both analyses were conducted. A total of 100 frames were considered for the calculation of the binding free energies [44].

### 2.10. C-Immune Simulations

The final vaccine constructs immunogenic efficacy was evaluated with the help of *in silico* immune simulation, by using “C-immSim server 10.1 [45]”. The vaccine's potential to interact with the immune system can be predicted in this method (http://tools.iedb.org/population/).

## 3. Results

### 3.1. Complete Genome Extraction and Subtractive Proteomics Analysis

**Figure 2:**
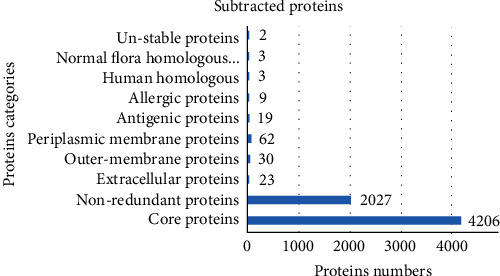
A number of proteins and their categories.

Two fully sequenced genomes of *C. valvarum* bacterium were retrieved from NCBI. BPGA analysis revealed that the fully sequenced genome consisted of 4206 core sequences. The core sequence was further considered for the selection of good vaccine targets. CD-HIT analysis predicted 2027 nonredundant proteins. The nonredundant proteins were processed for subcellular localization analysis. In subcellular localization analysis, 115 proteins were predicted in subcellular localized regions. Among total subcellular localized proteins, 23 were extracellular, 30 proteins were predicted in the outer-membrane region, and 62 proteins were predicted in the periplasmic membrane region. Nineteen subcellular localized proteins were expected to be antigenic, 9 antigenic proteins were predicted to be allergenic, and 2 of the remaining 10 proteins were predicted to be unstable having >100 MW. Among the 8 filtered proteins, 3 proteins were similar to humans, and 3 proteins were homologs to normal flora. Overall categories and numbers of subtracted proteins are presented in Figure 2.

### 3.2. Epitope Mapping Phase

After applying several subtractive filters, only two proteins TonB-dependent siderophore receptor and hypothetical protein were selected as vaccine candidates. From the first protein (TonB-dependent siderophore receptor), 10 different epitopes with various lengths were predicted, while from protein 2 (hypothetical protein), only 9 epitopes were predicted. The predicted epitopes are tabulated in Table 1.

### 3.3. T-Cell Epitope Prediction

Predicted B-cell epitopes were used to predict T-cell epitopes “MHC-I and II epitopes.” The predicted epitopes were ranked on the basis of lower percentile score. T-cell epitopes are tabulated in Table 2.

### 3.4. Multiepitope Construction and Processing

The predicted T-cell peptide was evaluated for antigenicity, allergenicity, and water solubility. Antigenic, water-soluble, and nonallergenic epitopes were shortlisted for vaccine design. The shortlisted epitopes are tabulated in Table 3.

**Figure 3:**
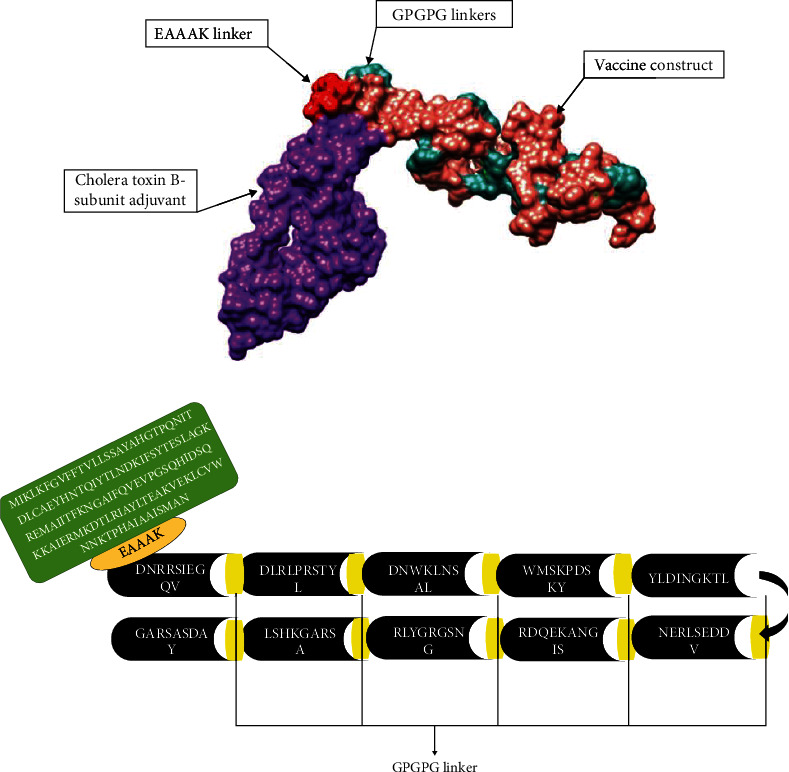
(a) 3D model of vaccine; (b) schematic representation of model vaccine.

In the multiepitope vaccine designing phase, the filtered epitopes were connected by “GPGPG” linkers. Additionally, the vaccine was bound to cholera toxin-B subunit adjuvant for boosting immune response. Physiochemical properties of the vaccine construct were analyzed. The server predicted that the vaccine construct comprises 272 amino acids with a molecular weight of 28646.22 and an instability index of 32.67. The VaxiJen 2.0 server predicted that the vaccine construct is probable antigenic with a 0.8925 antigenicity score. The 3D structure was modeled as presented in Figure 3(a), while the schematic representation of the multiepitope vaccine construct is shown in Figure 3(b).

### 3.5. Validation of Model Stability

The Ramachandran plot analysis shows that favourable areas are occupied by 182 (87.9%) of the residues in the protein model. Additionally, it was found that just 0% of residues were in banned or outline boundaries and that 11.6% (24) of residues were present in allowed regions. The overall number of residues (272), the number of glycine residues (40), the number of proline residues (23), and the number of end residues (2) were all estimated via the PROCHECK service. The overall quality factor of the vaccine construct was 44.4 calculated by the ERRAT tool.

### 3.6. Disulfide Engineering *In Silico* Codon Optimization and Loop Refinement

**Figure 4:**
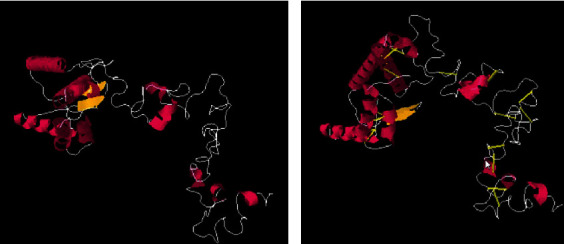
Mutant and original structure of model vaccine.

In disulfide engineering, a total number of sixteen pairs of amino acid residues were considered to make disulfide bonds: Ile2-glu125, chi3 value 111.32, and energy value 3.21; Leu14-asp28, chi3 value -65.84, and energy value 6.81; Ala19-ile38, chi3 value 89.41, and energy value 2.28; Thr27-his34, chi3 -89.18, and energy value 3.29; Thr36-tyr39, chi3 value 116.98, and energy value 3.86; Lys129-asn136, chi3 value -101.93, and energy value 5.9; Ser156-pro161, chi3 value -104.16, and energy value 6.49; Thr157-trp167, chi3 value 98.41, and energy value 2.47; Tyr158-pro161, chi3 value 114.52, and energy value 4.81; Ala172-gly176, chi3 value 69.34, and energy value 4.84; Gly190-pro203, chi3 value 105.85, and energy value 2.6; Leu194-gly198, chi3 value 93.64, and energy value 3.94; Gly198-leu201, chi3 value -61.97, and energy value 5.92; Thr200-pro217, chi3 value 110.96, and energy value 4.06; Gly202-asn207, chi3 value 88.96, and energy value 5.35; Pro203-leu210, chi3 value -92.84, and energy value 4.97. The mutated and original structure of the vaccine construct is mentioned in Figures 4(a) and 4(b).

Furthermore, the optimized sequence had a codon adaptation index (CAI) of 0.973897328694206, indicating an effective expression system in the *E. coli* host with a GC content of 54.04411764705882. The modified codon sequence from the vaccine construct “ATGATCAAACTGAAATTTGGCGTCTTCTTCACCGTCCTGCTGTCTTCTGCTTACGCTCACGGTACCCCGCAGAACATCACCGACCTGTGCGCTGAATACCACAACACC.

**Figure 5:**
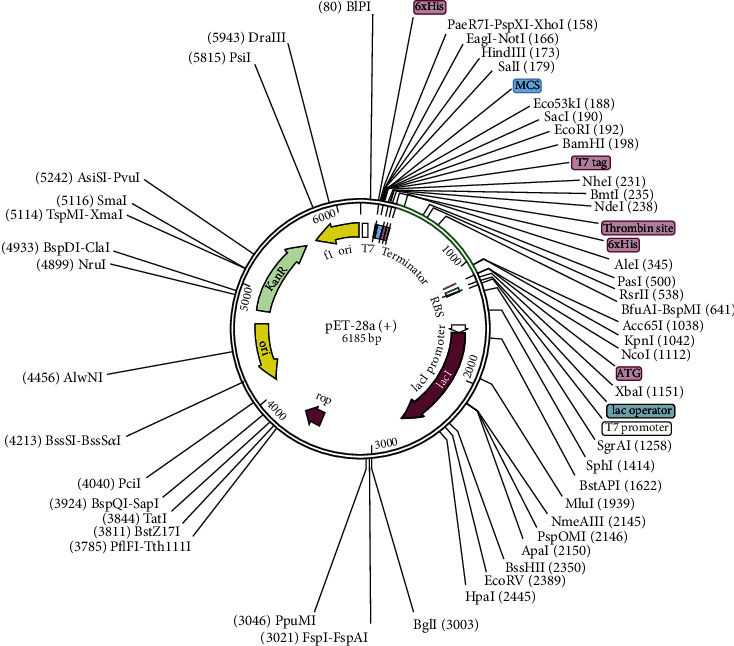
Cloned pET28a (+) vector.

AGATCTACACCCTGAACGACAAAATCTTCTCTTACACCGAATCTCTGGCTGGTAAACGTGAAATGGCTATCATCACCTTCAAAAACGGTGCTATCTTCCAGGTTGAAGTTCCGGGTTCTCAGCACATCGACTCTCAGAAAAAAGCTATCGAACGTATGAAAGACACCCTGCGTATCGCTTACCTGACCGAAGCTAAAGTTGAAAAACTGTGCGTTTGGAACAACAAAACCCCGCACGCTATCGCTGCTATCTCTATGGCTAACGAAGCTGCTGCTAAAGAAGCTGCTGCTAAAGACAACCGTCGTTCTATCGAAGGTCAGGTTGGTCCGGGTCCGGGTGACCTGCGTCTGCCGCGTTCTACCTACCTGGGTCCGGGTCCGGGTGACAACTGCTGCGTCTGCCGCGTTCTACCTACCTGGGTCCGGGTCCGGGTGACAACTGGAAACTGAACTCTGCTCTGGGTCCGGGTCCGGGTTGGATGTCTAAACCGGACTCTAAATACGGTCCGGGTCCGGGTTACCTGGACATCAACGGTAAAACCCTGGGTCCGGGTCCGGGTAACGAACGTCTGTCTGAAGACGACGTTGGTCCGGGTCCGGGTCGTGACCAGGAAAAAGCTAACGGTATCTCTGGTCCGGGTCCGGGTCGTCTGTACGGTCGTGGTTCTAACGGTGGTCCGGGTCCGGGTCTGTCTCACAAAGGTGCTCGTTCTGCTGGTCCGGGTCCGGGGGTGCTCGTTCTGCTTCTGACGCTTAC” was then inserted in *E. coli* expression vector PET28a (+), as shown in Figure 5. In loops refinement, 10, the model was refined as the data of loops refinement is tabulated in Table S4.

### 3.7. Molecular Docking Analysis

**Figure 6:**
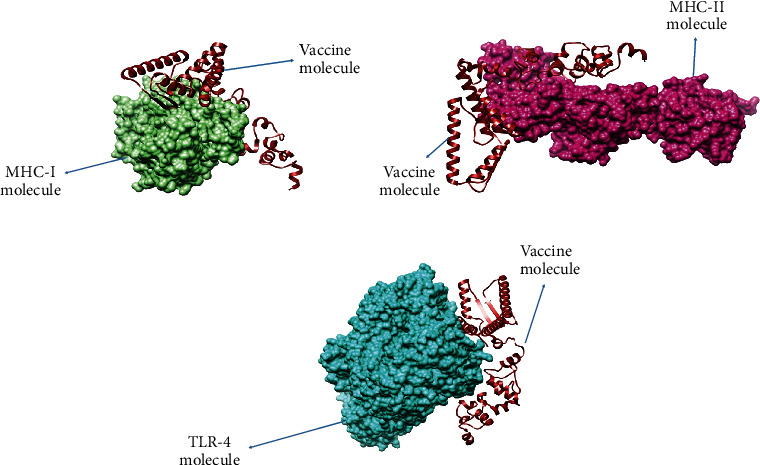
Docked confirmation of vaccine and selected immune cell receptors.

Interaction of vaccine with host immune cells is vital for inducing immune responses, these interactions were analyzed through molecular docking analysis, and the docking server generated 20 docked complexes as mentioned in supplementary Tables S1-S4. The PDBsum analysis of vaccine 1 revealed 16 hydrogen bonds and 1 salt bridge. Moreover, 42 interface residues were discovered in vaccine 1, covering an interface area of 2,502 (A2), compared to 45 MHC-I interface residues, which covered an area of 2,319 (A2). PDBsum estimated two salt bridges and eight hydrogen bonds for vaccine 2. Additionally, interactions between 26 and 36 MHC-II and vaccine 2 residues occurred over 1,997 and 1,832, respectively. Similar to vaccine 2, PDBsum predicted 14 hydrogen bonds and one salt bridge for vaccine 3. Additionally, 27 and 22 residues from vaccine 3 and TLR-4, covering 1,228 and 1,271, respectively, interacted with one another. Further, the docking results were refined, and from refined complexes, top 1 complexes in each case of receptors were considered for the simulation study. The top complexes are tabulated in Table 4 while the docked 3D confirmation of docked complexes is mentioned in Figures 6(a)–6(c).

### 3.8. Molecular Dynamic Simulation

**Figure 7:**
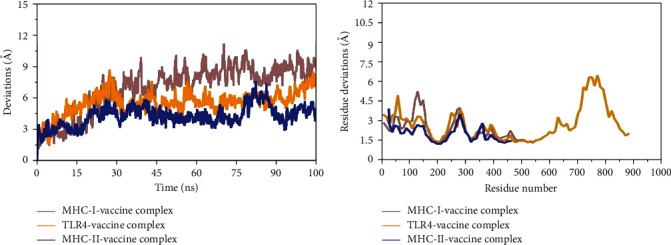
Statistical graphs of the simulation trajectories.

Molecular dynamic simulation analysis was done for analyzing the movement of the macromolecules docked complexes [46]. MD simulation analysis was carried out for vaccine-MHC-I, MHC- and TLR-R for 100 ns seconds. In MD simulation, root mean square fluctuation (RMSF) and root-mean-square deviation (RMSD) analysis were performed. We observed, in the RMSD, that the vaccine and MHC-II molecule have stable binding affinity as it showed little deviation followed by TLR-4 and vaccine and MHC-I with vaccine as presented in Figure 7(a). Subsequently, RMSF analysis was done to evaluate residue level fluctuation. The RMSF analysis found lower fluctuations between docked complexes as presented in Figure 7(b). Overall, in the whole period of simulation, no drastic changes were observed in docked complexes.

### 3.9. Normal Mode Simulation Analysis

**Figure 8:**
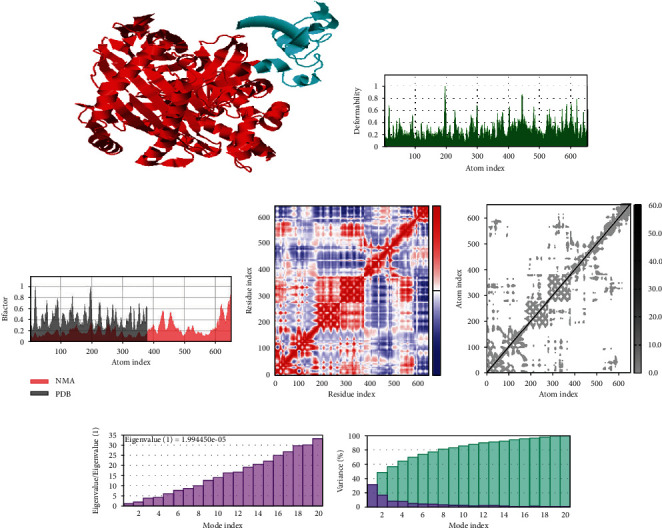
Statistical analysis of normal mode simulation graphs analysis of vaccine-MHC-I molecule obtained from IMODS server.

**Figure 9:**
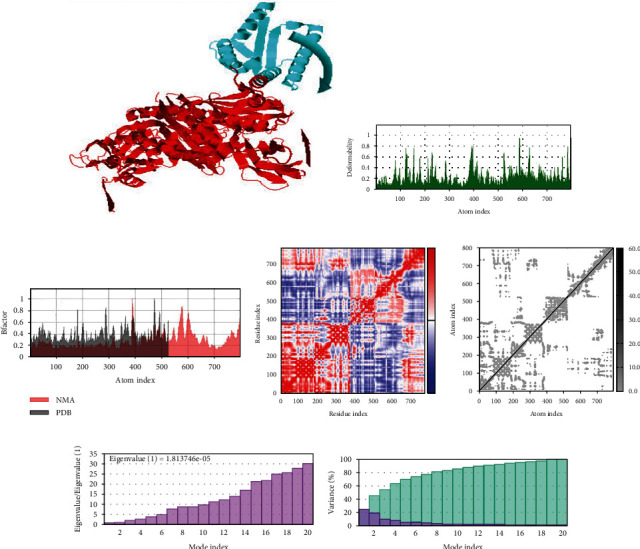
Statistical analysis of normal mode simulation graphs analysis of vaccine-MHC-II molecule obtained from iMODS server.

**Figure 10:**
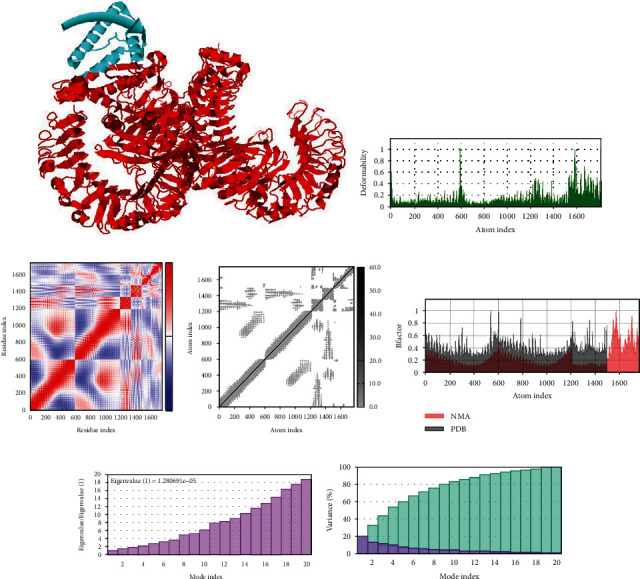
Statistical analysis of normal mode simulation graphs analysis of vaccine-TLR-4 molecule obtained from IMODS server.

In normal mode simulation analysis, the vaccine-immune cell receptor docked complexes are further stimulated for binding stability analysis. Direction of the residues is represented by the arrow in Figures 8–10(a) in which the vaccine construct is shown by red color, while immune cell receptors are represented by blue color. In beta factor mobility analysis, both the vaccine and receptors are found to be mobile proteins that can allow interaction between docked molecules, and the beta factor mobility of vaccine-MHC-I, MHC-II, and TLR-4 is represented in Figures 8–10(b). Next, the experimental B-factor is taken from the corresponding PDB field and the calculated from NMA is obtained by multiplying the NMA mobility by (8*π*^2^). Be aware that many PDB files of averaged NMR models contain no B-factors (actually, the B-factor column gives an averaged RMS) as the beta factor and NMA of vaccine and MHC-I, MHC-II, and TLR-4 are presented in the following 8, 9 and 10C. The covariance map of vaccine-MHC-I, II, and TLR-4 is shown in Figures 8–10(d), which represents the coupling between pairs of residue, either they are correlated, uncorrelated, or anticorrelated motions which are represented by red, white, and blue, respectively. In the elastic network analysis define pairs of residues connected by springs, each dot in the graph represents one spring between the corresponding pair of atoms. Dots are colored according to their stiffness, the darker grays indicate stiffer springs and vice versa as presented in Figures 8–10(e). The eigenvalue is directly related to the energy required to deform the structure, the lower the eigenvalue, the easier the deformation, the eigenvalue associated to each normal mode shows the stiffness, and the eigenvalue and mode index of vaccine- MHC-I, MHC-II, and TLR-4 are presented in Figures 8–10(f). Furthermore, in variance analysis, the inversely related to the eigenvalue was analyzed in Figures 8–10(g) the colored bars show the individual (red) and cumulative (green) variances in the case of vaccine with MHC-I, MHC-II, and TLR-4, respectively.

### 3.10. Binding Energy Estimation

In binding energies estimation net binding energies were calculated through Molecular mechanics with generalised Born and surface area solvation (MMGBSA) and Molecular Mechanics Poisson-Boltzmann Surface Area (MMPBSA), the MMGBSA calculated Delta Total net binding energies for “TLR-4-Vaccine Complex, MHC-I-Vaccine Complex and MHC-II-Vaccine Complex -94,-78 and -76”, respectively. The MM-PBSA analysis calculated -97,-61, and -72 net binding energy for a vaccine with TLR-4, a vaccine with MHC-I-, and vaccine with MHC-II- as mentioned in the following Table 5.

### 3.11. Chemical Interaction of Vaccine to Immune Cell Receptors

Proper immune responses are not produced if there is no interaction between the host immune cells and the vaccine. Using a protein-peptide molecular docking technique, it was discovered how chemically the vaccine design interacted with toll-like receptor-4, major histocompatibility complex-I, and major histocompatibility complex-II. Utilizing the UCSF chimera tool, specific amino acid residues base interactions between MCH-I, MCH-II, and TLR-4 were examined. The interactions include both hydrophobic and hydrophilic interactions. Van der Waals, hydrogen bonds, and salt bridge interactions are among the close-proximity interactions that are taking place. One of the several proteins known as toll-like receptors (TLRs) that aid in the initiation of both acquired and adaptive immune responses is toll-like receptor 4, which is mainly expressed on immune cells. The interactive amino acid with immune cell receptors is tabulated in Table 6.

### 3.12. *In Silico* Immune Stimulation

**Figure 11:**
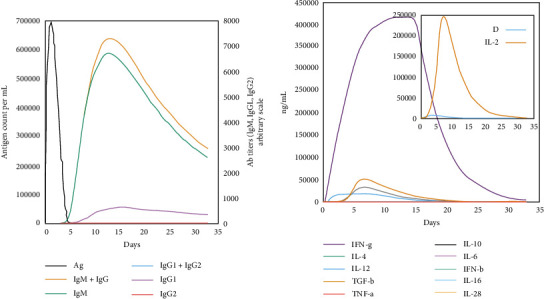
Production of different antibody titers (a) in response to the vaccine antigen. (b) The concentration of the cytokines and interleukins against the vaccine antigen.

The host immunological simulation carried out by the C-ImmSim server analyzed the host immune response to the vaccine. The server predicted that the designed vaccine construct properly induce an immune response in the form of different antibodies and other cytokines. Antibody response toward vaccine is presented in Figure 11(a), while other cytokines and interferon are presented in Figure 11(b). Furthermore, toward the vaccine, different interleukins (IL-4 and IL-12) and transforming growth factor-beta (TGF*β*) are also observed in different levels.

### 3.13. Population Coverage Analysis of Selected Epitopes

**Figure 12:**
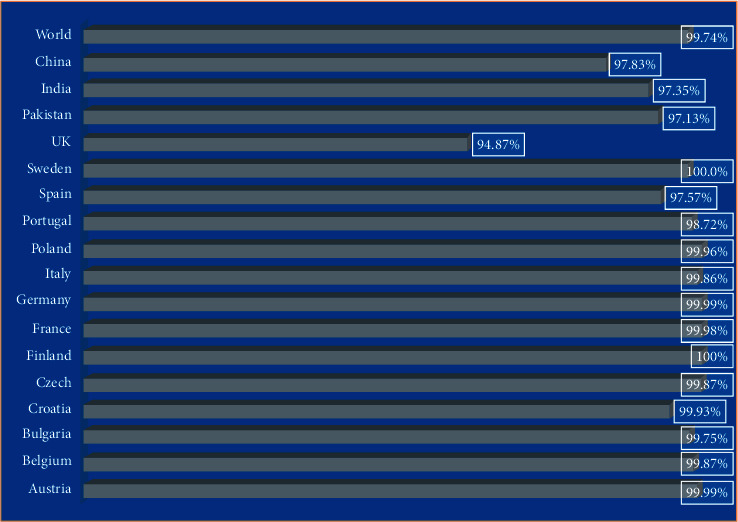
Population coverage analysis covered by conserved selected antigenic epitopes; the data is generated by the immune epitopes database online web server.

In population coverage analysis, the selected epitopes were tested for population coverage analysis using online immune epitope database; in this analysis, the conserved selected epitopes predicted that the selected epitopes can cover 99.75% population of the world, 97.83 population of China, and 97.35% of India followed by Pakistan, and other countries are mentioned in Figure 12; therefore, the selected epitopes were used in the designing of multiantigenic chimeric vaccine construct against the target pathogen.

## 4. Discussions

Although *C. valvarum* infection may not currently pose a life-threatening threat, medical attention to this opportunistic disease is constantly growing [8]. Several approaches are still present to manage bacterial infections by developing preventive measures and vaccinations before the occurrence of infection [47]. *C. valvarum* is unquestionably occurring as a result of the widespread and unchecked use of antibiotics. The pan-genome analysis is an alternative and the reverse of the pasture vaccinology approach for designing multiepitope constructs against several bacterial pathogens [48]. In addition, the genomic data offered important new information. Several genetic alterations occur during infection due to variances in genome large size and functional genes' developed genetic structure. *C. valvarum* strains differ physiologically and genomically [49]. Along with analyzing the *C. valvarum* genome, our goal was to create a multiepitope vaccine to fight this opportunistic infection; as in a previously conducted study, *in silico* vaccine was designed against *Enterococcus mundtii* [29]. To overcome the limitations of this method, other criteria were considered when selecting antigens for vaccine formulation, such as epitope antigenicity, physicochemical stability, nonallergenicity, and nontoxicity. Using epitope mapping and selection, probable antigenic epitopes were selected for vaccine design. As in a previous study, several epitopes were mapped and filtered for multiepitope vaccine construction against *Morganella morganii* to induce a strong immune response against the target pathogen [36]. The conserved and antigenic nature of the discovered T-cell epitopes raises the possibility that they could play a significant role in a designed vaccine. To avoid this, a high-quality and stable three-dimensional structure of the vaccine model was modeled. This study is also aimed at generating both T-cell and B-cell immune activations. Hence, the vaccine comprises both B and T-cell epitopes because T-cell responses not only have a lengthy half-life but can also prevent antigenic drift. The designed vaccine has high thermodynamic viability, stability, hydrophilicity, and expression capacity. No adverse responses are anticipated because the multiepitope vaccine is nonallergenic [39].Molecular docking and simulation approaches were utilized to evaluate the binding affinity and movement of docked molecules for confirmation of binding stability, similar to a previous study conducted for multiepitope vaccine designing [27], as our findings and the previous study findings support result of each other.

## 5. Conclusion

Finally, employing a probable vaccine target within *C. valvarum* core proteome, we used computational tools to create an *in silico* vaccine against *C. valvarum*. The discovered proteins were utilized for epitope prediction and to elicit immune responses. The model vaccine has good binding capacity as it is crucial for generating the activation of the immune system. The movement of docked molecules and the interaction of immune cell receptors were further validated through a simulation study. The model vaccine showed the best immunogenicity and was able to induce a proper immune response against the target pathogen. These and other limitations call for further development and investigation in future research efforts. Vaccine development proteins were subjected to highly strict selection and filtering criteria. However, these selection criteria of vaccine targets are required for experimental validation. In conclusion of this study, by applying several immunoinformatics approaches, we observed that our proposed vaccine construct could induce a proper immune response against *C. valvarum* and can reduce the infection caused by the said pathogen. However, our study is based on computational approaches; hence, experimental validation is strongly recommended. The vaccine candidate could speed the vaccine development process during the formulation of a vaccine against the target pathogen.

## Figures and Tables

**Table 1:** Predicted B-cell epitopes.

Selected proteins	Predicted epitopes
TonB-dependent siderophore receptor	RPTADNRRSIEGQVDRFGKVR
	DVKGRDLRLPRSTYLGASWNRSTYHK
	DHQFNDNWKLNSALEYKH
	YVPQRSNVSASGTVS
	REKFDNTWHGKKIAGEYNIFRWQGTEIAQPADWNALPEEVRHT
	WQQHQHLSWMSKPDSKYGKGRL
	FKYSGDYLDINGKTL
	QPIDTWKGIDPATGAVREWQRGDRSIYTPVRLQ
	YTSTAATKTITRNGKGVDFSQHTP
	MQSKSSPITVDGNKHYL

Hypothetical protein	Predicted epitopes
	QNAPPPSRALPVDIDRGNERLSEDDVGKNVRLPQVPAVKPAAPTTLPSDAQAAKEDVVYTPEELVNNPEQ
	KLRSGDVSEADRKTL
	IHDNNVNNVAPKGTRFRLSNGRSLESNRDQEKANGIS
	SGSYYFHKRNYNDVTT
	KRLYGRGSNGDNKLHAYSN
	EFSYDKYVRTYDYLDGK
	LSHKGARSASDAYD
	YGKRYSKGVDFFNIDREDK
	FSYSKIDSNNRFYSYDAS

**Table 2:** T-cells epitopes and percentile score.

MHC-I	Percentile score	MHC-II	Percentile score
RSIEGQVDRF	0.09	DNRRSIEGQVDRF	17
DNRRSIEGQV	12	DNRRSIEGQVDRF	17
RPTADNRRSI	0.04	RPTADNRRSIEGQV	44
NRRSIEGQV	9.8	RPTADNRRSIEGQV	44
RSIEGQVDRF	0.09	RSIEGQVDRFGKVR	26
QVDRFGKVR	1.4	RSIEGQVDRFGKVR	26
TYLGASWNR	0.03	STYLGASWNRSTYH	6.1
ASWNRSTYH	0.63	STYLGASWNRSTYH	6.1
DVKGRDLRL	0.08	DVKGRDLRLPRSTYL	9.2
DLRLPRSTYL	0.12	DVKGRDLRLPRSTYL	9.2
KLNSALEYK	0.02	NWKLNSALEYK	0.29
NWKLNSALEY	1.2	NWKLNSALEYK	0.29
HQFNDNWKL	0.06	DHQFNDNWKLNSAL	9.8
DNWKLNSAL	1.5	DHQFNDNWKLNSAL	9.8
NVSASGTVS	10	YVPQRSNVSASGTVS	5.2
DWNALPEEV	1.7	QPADWNALPEEV	1.4
QPADWNALP	4.1	QPADWNALPEEV	1.4
IFRWQGTEI	1.4	YNIFRWQGTEIAQP	4.2
WQGTEIAQP	11	YNIFRWQGTEIAQP	4.2
WHGKKIAGE	31	WHGKKIAGE	21
KFDNTWHGK	1.1	REKFDNTWHGKKIA	27
NTWHGKKIA	2.5	REKFDNTWHGKKIA	27
HQHLSWMSK	0.05	HQHLSWMSKPDSK	11
LSWMSKPDSK	0.85	SWMSKPDSKYGKGRL	48
WMSKPDSKY	0.08		
DSKYGKGRL	2.6	SWMSKPDSKYGKGRL	48
HQHLSWMSK	0.05	WQQHQHLSWMSKPDS	20
WQQHQHLSW	0.59	WQQHQHLSWMSKPDS	20
LSWMSKPDS	21	FKYSGDYLDINGKTL	11
YLDINGKTL	0.14		
FKYSGDYLDI	3.1	FKYSGDYLDINGKTL	11

**Table 3:** Selected epitopes for multiepitope vaccine designing.

Selected epitopes	Predicted ic50 value (nm)	Antigenicity	Allergenicity	Toxicity	Water solubility
DNRRSIEGQV	7.242	1.3151	Nonallergic	Nontoxic	Good water solubility
DLRLPRSTYL	7.558	0.7153			
DNWKLNSAL	6.983	1.1081			
WMSKPDSKY	7.536	0.9954			
YLDINGKTL	6.837	1.4469			
NERLSEDDV	7.174	1.1854			
RDQEKANGIS	8.432	1.0961			
RLYGRGSNG	6.764	1.9978			
LSHKGARSA	7.07	1.3193			
GARSASDAY	7.356	0.9841			

**Table 4:** Top-docked complexes with their least binding energy score were selected for simulation.

Top-docked complexes	Global energy	Attractive VdW	Repulsive VdW	ACE	HB
Vaccine-MHC-I	6.89	-4.75	0.92	2.52	16
Vaccine-MHC-II	12.75	-4.94	1.02	1.90	8
Vaccine-TLR-4	-19.51	-12.63	0.91	2.80	14

**Table 5:** Net binding-free energy calculation.

Energy parameter	TLR-4 and vaccine	MHC-I- and vaccine	MHC-II-and vaccine
*MM-GBSA*			
VDWAALS	-78.00	-60.00	-65.00
EEL	-45.00	-39.00	-31.00
Delta G gas	-123	-99	-96
Delta G solv	29.00	21.00	20.00
Delta total	-94	-78	-76
*MM-PBSA*			
VDWAALS	-78.00	-60.00	-65.00
EEL	-45.00	-39.00	-31.00
Delta G gas	-123	-99	-96
Delta G solv	26.00	38.00	24.00
Delta total	-97	-61	-72

**Table 6:** Immune cell receptors and interactive amino acid residues.

Immune cells receptors	Interactive residues
Vaccine-MHCI	Gln180, Lys176, Ala211, Pro210, Glu232, Tyr209, Thr233, Glu177, Thr178, Pro235, Ile7 Tyr26, Pro5, Asn174, Phe30, Thr86, Asp53, Leu87, Ile35, Glu55.
Vaccine-MHC-II	Arg4, Asp17, Val42, Leu14, Phe7, Glu46, Pro81, Glu47, Glu30, Leu38, Val116, His167, His149, Asn118, Val165, Thr129, Cys107, Pro87, Arg146, Leu138.
Vaccine-TLR-4	Leu434, Lys89, Tyr79, Cys390, Glu150, Ile412, Asn86, Gln436, Ile52, Asn433, Ser120, Val411, Glu439, Gln436, Ser441, Glu494, Leu470, Lys477, Asn468, Val442.

## Data Availability

The data presented in this study are available within the article.

## Supplementary Materials

Supplementary Materials Supplementary Table S1: docked solution of vaccine with MHC-I generated by Patch dock server. Supplementary Table S2: docked solution of vaccine with MHC-II generated by Patch dock server. Supplementary Table S3: docked solution of vaccine with a toll-like receptor-4 (TLR-4) generated by Patch dock server. Supplementary Table S4: top 10 refine model generated by refine 2 services in GALAXY webserver.
