# Supplementary material for: Development of a Novel Vaccine Candidates against Cardiobacterium valvarum through Reverse Vaccinology and Computational Approaches
Source: Biomed Res Int. 2023 Jun 28;2023:6325568. doi: 10.1155/2023/6325568 (PMC10322295; doi:10.1155/2023/6325568)
Supplement: Supplementary Materials — Supplementary Table S1: docked solution of vaccine with MHC-I generated by Patch dock server. Supplementary Table S2: docked solution of vaccine with MHC-II generated by Patch dock server. Supplementary Table S3: docked solution of vaccine with a toll-like receptor-4 (TLR-4) generated by Patch dock server. Supplementary Table S4: top 10 refine model generated by refine 2 services in GALAXY webserver. [file 6325568.f1.docx]

**Supplementary Table S1**. Docked solution of vaccine with MHC-I generated by Patch dock server.

| **Solution No** | **Score** | **Area** | **Atomic Contact Energy (Kj/mol)** |
| --- | --- | --- | --- |
| 1 | 20654 | 3183.7 | 304.80 |
| 2 | 19512 | 2509.3 | 311.27 |
| 3 | 17904 | 2337.9 | 375.61 |
| 4 | 17818 | 2350.5 | 291.20 |
| 5 | 17400 | 2269.2 | 382.84 |
| 6 | 17204 | 2558.7 | 473.86 |
| 7 | 17014 | 2536.2 | 387.57 |
| 8 | 16788 | 2914.8 | -23.21 |
| 9 | 16782 | 2377.3 | 416.76 |
| 10 | 16740 | 2610.8 | 313.86 |
| 11 | 16704 | 2520.3 | 188.05 |
| 12 | 16674 | 2892.2 | 396.21 |
| 13 | 16664 | 2167.4 | 436.38 |
| 14 | 16662 | 2489.1 | 474.81 |
| 15 | 16638 | 2432.8 | 323.60 |
| 16 | 16606 | 2503.1 | 472.48 |
| 17 | 16244 | 3088.7 | 136.97 |
| 18 | 16128 | 2184.8 | 345.54 |
| 19 | 16094 | 2999.7 | 188.24 |
| 20 | 16074 | 2413 | 427.86 |

**Supplementary Table S2**. Docked solution of vaccine with MHC-II generated by Patch dock server.

| **Solution No** | **Score** | **Area** | **Atomic Contact Energy (Kj/mol)** |
| --- | --- | --- | --- |
| 1 | 20268 | 2610.9 | 109.72 |
| 2 | 19242 | 2412.4 | 366.62 |
| 3 | 19136 | 2885.3 | 59.35 |
| 4 | 19008 | 3439.6 | 139.93 |
| 5 | 18436 | 2357.6 | 248.34 |
| 6 | 18378 | 3266.6 | 207.84 |
| 7 | 18300 | 3294.9 | 117.24 |
| 8 | 18280 | 2170.2 | 336.14 |
| 9 | 18274 | 3429.8 | 420.77 |
| 10 | 18018 | 3661.4 | 410.34 |
| 11 | 17962 | 2813.2 | 217.68 |
| 12 | 17930 | 2311 | 112.54 |
| 13 | 17382 | 2986.3 | 417.25 |
| 14 | 17332 | 3053.3 | 133.99 |
| 15 | 17316 | 2956.5 | -183.61 |
| 16 | 17182 | 3021 | 448.65 |
| 17 | 17158 | 2825.2 | 396.61 |
| 18 | 17154 | 2183.4 | 269.01 |
| 19 | 17026 | 2857.1 | 446.10 |
| 20 | 17020 | 2684.1 | 434.31 |

**Supplementary Table S3**. Docked solution of vaccine with tool like receptors 4 (TLR-4) generated by Patch dock server.

| **Solution No** | **Score** | **Area** | **Atomic Contact Energy (Kj/mol)** |
| --- | --- | --- | --- |
| 1 | 19870 | 3420.6 | 99.37 |
| 2 | 19352 | 3004.5 | 13.16 |
| 3 | 19108 | 2986.2 | 346.22 |
| 4 | 18766 | 3456.6 | 365.32 |
| 5 | 18380 | 2300.6 | 442.17 |
| 6 | 18288 | 2889.1 | 469.17 |
| 7 | 18258 | 2821.9 | 264.89 |
| 8 | 18242 | 2750.1 | 492.66 |
| 9 | 17694 | 2864.3 | 275.62 |
| 10 | 17626 | 2136.9 | 443.96 |
| 11 | 17500 | 2787.4 | 448.18 |
| 12 | 17378 | 3157.3 | 373.72 |
| 13 | 17376 | 2687.9 | 405.28 |
| 14 | 17342 | 2712.3 | 209.11 |
| 15 | 17244 | 3580.5 | -89.14 |
| 16 | 17054 | 3317.3 | 351.03 |
| 17 | 17038 | 2375.8 | 471.94 |
| 18 | 17002 | 2416.2 | 423.86 |
| 19 | 16966 | 2760.6 | 120.97 |
| 20 | 16696 | 2655.1 | 283.29 |

**Supplementary Table S4.** Top-10 refine model generated by refine 2 service in GALAXY webserver.

| **Model** | **RMSD** | **MolProbity** | **Clash score** | **Poor rotamers** | **Rama favored** | **GALAXY energy** |
| --- | --- | --- | --- | --- | --- | --- |
| Initial | 0.000 | 3.630 | 96.9 | 8.0 | 91.9 | 29663.52 |
| MODEL 1 | 0.934 | 1.436 | 2.3 | 0.5 | 93.3 | -4302.34 |
| MODEL 2 | 1.387 | 1.055 | 0.7 | 0.0 | 95.2 | -4300.14 |
| MODEL 3 | 1.085 | 1.282 | 1.6 | 0.0 | 94.4 | -4297.71 |
| MODEL 4 | 1.007 | 1.341 | 2.5 | 0.5 | 95.6 | -4289.52 |
| MODEL 5 | 0.983 | 1.201 | 1.1 | 1.0 | 94.4 | -4286.90 |
| MODEL 6 | 0.987 | 1.070 | 1.1 | 0.5 | 96.3 | -4280.47 |
| MODEL 7 | 0.912 | 1.223 | 1.4 | 0.5 | 94.8 | -4279.82 |
| MODEL 8 | 1.054 | 0.969 | 0.7 | 0.5 | 96.3 | -4274.86 |
| MODEL 9 | 0.908 | 1.156 | 1.1 | 1.0 | 95.2 | -4274.62 |
| MODEL 10 | 0.935 | 1.238 | 1.1 | 0.5 | 93.7 | -4272.69 |
